# Effect of cement spacer on fit accuracy and fracture strength of 3-unit and 4-unit zirconia frameworks

**DOI:** 10.1186/s12903-024-04341-3

**Published:** 2024-05-21

**Authors:** Noha Morsy, Mona Mohamed Ghoneim, Yomna Ibrahim

**Affiliations:** 1 Department of Conservative Dentistry, Faculty of Dentistry, Alexandria University, Alexandria, Egypt; 2 Department of Dental Biomaterials, Faculty of Dentistry, Alexandria University, Alexandria, Egypt

**Keywords:** Cement spacer, Fit, Fracture, Zirconia

## Abstract

**Background:**

Cement spacer is essential for compensating deformation of zirconia restoration after sintering shrinkage, allowing proper seating and better fracture resistance of the restoration. Studies assessing the effect of cement spacer on fit accuracy and fracture strength of zirconia frameworks are missing in the literature. Therefore, the aim of this study was to evaluate the effect of different cement spacer settings on fit accuracy and fracture strength of 3-unit and 4-unit zirconia frameworks.

**Methods:**

Sixty standardized stainless-steel master dies were manufactured with 2 prepared abutments for fabricating 3-unit and 4-unit zirconia frameworks. The frameworks were assigned into 6 groups (*n* = 10) according to cement spacer setting (30 μm, 50 μm, and 80 μm) as follows: 3-unit frameworks; 3u-30, 3u-50, 3u-80, and 4-unit frameworks; 4u-30, 4u-50, and 4u-80. The frameworks were assessed for fit accuracy with the replica method. The specimens were cemented to their corresponding dies, and the fracture strength was measured in a universal testing machine. The Weibull parameters were calculated for the study groups and fractured specimens were inspected for failure mode. Two-Way ANOVA followed by Tukey test for pairwise comparison between study groups (α = 0.05).

**Results:**

The cement spacer had a significant effect on both fit accuracy and fracture strength for 3-unit and 4-unit frameworks. The 50 μm spacer had significantly better fit accuracy followed by 80 μm, and 30 μm spacers. Both 50 μm and 80 μm spacers had similar fracture strength, and both had significantly better strength than 30 μm spacer.

**Conclusions:**

For both 3-unit and 4-unit zirconia frameworks, 50 μm cement spacer can be recommended over 30 μm and 80 μm spacers for significantly better fit accuracy and adequate fracture strength.

## Background

Zirconia has become a popular material for fabricating frameworks for posterior fixed partial dentures (FPDs), due to its satisfactory mechanical properties and biocompatibility [1, 2]. Presintered zirconia blanks for computer-aided designing-computer-aided milling (CAD-CAM) can be milled easily. However, the milled restoration undergoes sintering shrinkage of 20–25%, which may lead to framework deformation and jeopardize the adaptation and cement layer thickness. In addition, sintering shrinkage can induce bending stresses within the framework, that along with ununiform cement layer can reduce fracture resistance of the restoration [3–5].

Fit accuracy and fracture resistance are critical for durability of a zirconia FPD [6, 7]. A marginal gap of 120 μm and an internal gap of 200 μm were reported in the literature as the clinically acceptable limit [8, 9]. For fracture resistance, a posterior restoration was recommended to withstand a force of 1000 N to be suitable for clinical service [10]. The span length of a FPD is a significant factor affecting fit accuracy and fracture resistance of FPDs. Theoretically, a 4-unit zirconia FPD can display more sintering stresses and deformation compared to a 3-unit FPD due to greater zirconia volume and longer span length [11]. Previous studies evaluated marginal and internal adaptation, and fracture strength of 3-unit [7, 9, 12–17] and 4-unit zirconia FPDs [6, 11, 18–20], and the reported findings were variable.

The die spacer or cement spacer is essential for compensating deformation of zirconia restoration after sintering shrinkage, allowing proper seating and adequate fit accuracy. Moreover, a cement spacer can ensure a uniform cement layer with better fracture resistance of the restoration [5, 21]. Few studies in the literature have assessed the effect of cement spacer on fit accuracy and fracture strength of zirconia CAD-CAM restorations, and the results are not conclusive [22–27]. An in vitro study by Kale et al. [22] reported better marginal fit of zirconia crowns with a cement spacer of 50 μm compared to 30 μm and 40 μm. A similar study by Schriwer et al. [23] reported better fit accuracy with a spacer of 60 μm compared to 30 μm, with no significant effect of cement spacer on the fracture resistance. A systematic review study by Morsy et al. [24] reported improved fit accuracy with a cement spacer ≤ 50 μm while two review studies by Hasanzade et al. [25, 26] reported better fit accuracy with a cement spacer ≤ 30 μm. The authors are aware of one study in the literature reporting the effect of cement spacer on fit accuracy of zirconia 3-unit FPDs, the study reported better adaptation with 30 μm spacer compared to 45 μm [27]. No studies in the literature investigated the effect of cement spacer on fracture resistance of CAD-CAM zirconia FPDs. Therefore, this study was conducted to assess the effect of cement spacer on marginal and internal fit, and fracture resistance of 3-unit and 4-unit CAD-CAM zirconia frameworks. The null hypothesis was that the cement spacer would have no significant effect on fit accuracy or fracture resistance for both 3-unit and 4-unit zirconia frameworks.

## Methods

### Fabrication of master dies

Thirty standardized master dies were fabricated for 3-unit FPDs frameworks. The dies were fabricated from stainless-steel (316 L UNS S3 Alloy; Masteel) by using a numerical control machine (EMCO Turn 343; EMCO G.). The design of the dies was created on a software program (AutoCAD 2011; Autodesk). The dies consisted of 2 prepared abutments attached to a base with dimensions of 30 × 17 × 4.5 mm. The abutments had 10 mm diameter; 5 mm axial height; 10° axial taper;1 mm rounded shoulder margin; and axio-occlusal angle with curvature radius of 0.8 mm. The distance between the centre of the abutments was 17 mm. Another 30 stainless-steel master dies were fabricated for 4-unit FPDs frameworks with the same method as before with 27 mm distance between abutments [15, 19, 28]. All master dies (*n* = 60) were subjected to air abrasion with 100 μm alumina to create a matt surface.

### Fabrication of zirconia frameworks

The master dies were scanned with a laboratory scanner (Medit T710; Medit Corp.), and the scans were saved as STL files and exported to a CAD software program (exocad 2021; exocad GmbH) to design zirconia frameworks. The frameworks were designed according to the manufacturer recommendations with 0.5 mm thickness, a 9 mm^2^ rounded connectors. The 3-unit frameworks were assigned into 3 groups (*n* = 10) according to the cement spacer as follows: group 3u-30 with 30 μm cement spacer; group 3u-50 with 50 μm cement spacer; and group 3u-80 with 80 μm cement spacer. Similarly, the 4-unit frameworks were assigned into 3 groups (*n* = 10) according to the cement spacer as follows: group 4u-30 with 30 μm cement spacer; group 4u-50 with 50 μm cement spacer; and group 4u-80 with 80 μm cement spacer. For all designed frameworks, the cement spacer started 1 mm above the finish line. The sample size was calculated with a software package (G*power 3.1.9.6; Heinrich-Heine-Universität) assuming a study power of 0.80 and an alpha error 0.05 based on the results of Rodríguez et al. [16].

The designs for the frameworks were exported to a 5-axis milling machine (DWX-52D Plus; Roland DG Corp), and zirconia frameworks were milled and sintered according to the manufacturer recommendations from presintered zirconia blanks (XTCERA HT preshaded; Shenzhen Xiangtong Co.).

### Assessment of marginal and internal fit accuracy

**Fig. 1:**
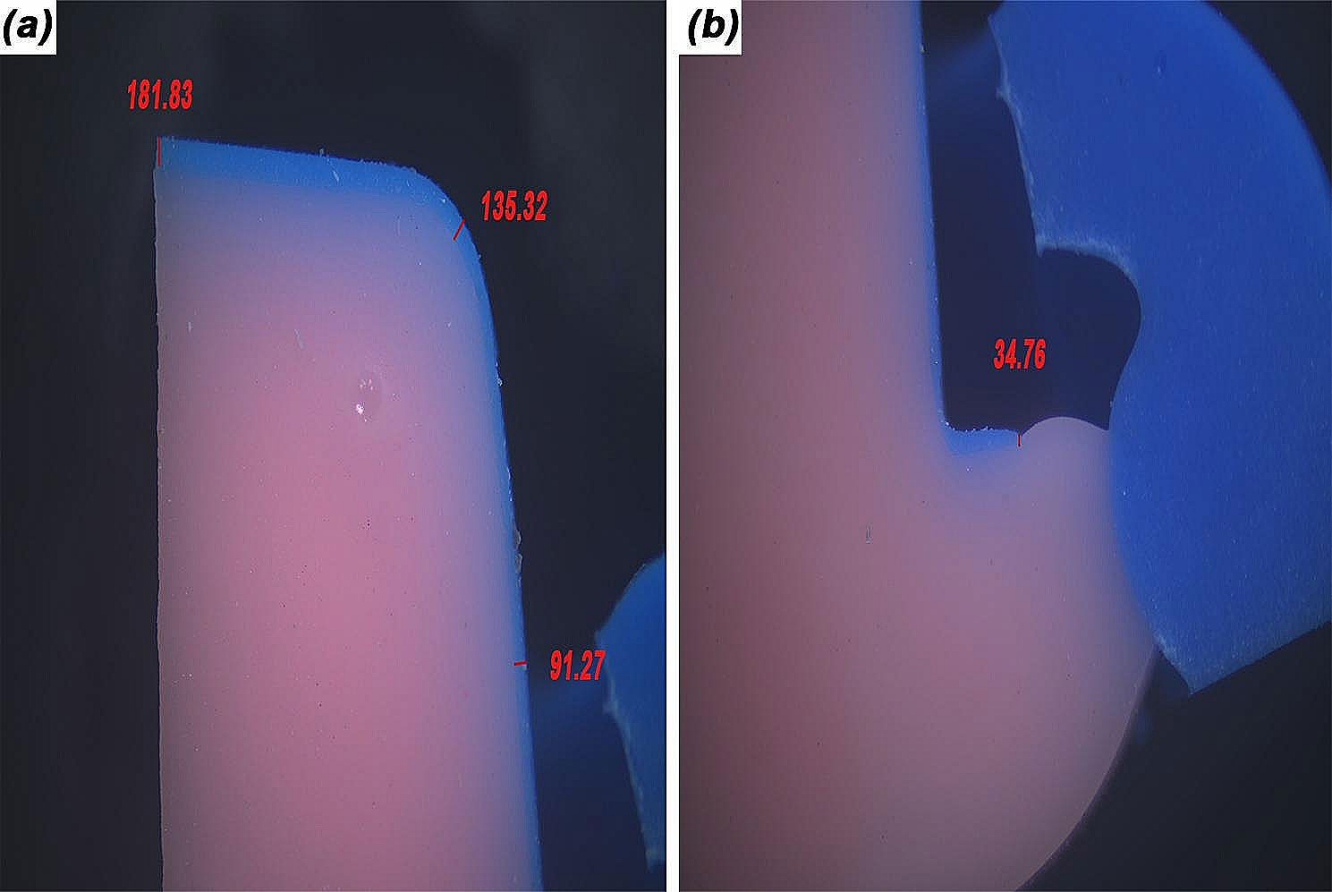
Measuring marginal and internal gaps with replica method. **A**, measuring internal gap at mid-occlusal, axio-occlusal, and mid-axial areas at ×40 magnification. **B**, measuring marginal gap at ×40 magnification

**Fig. 2:**
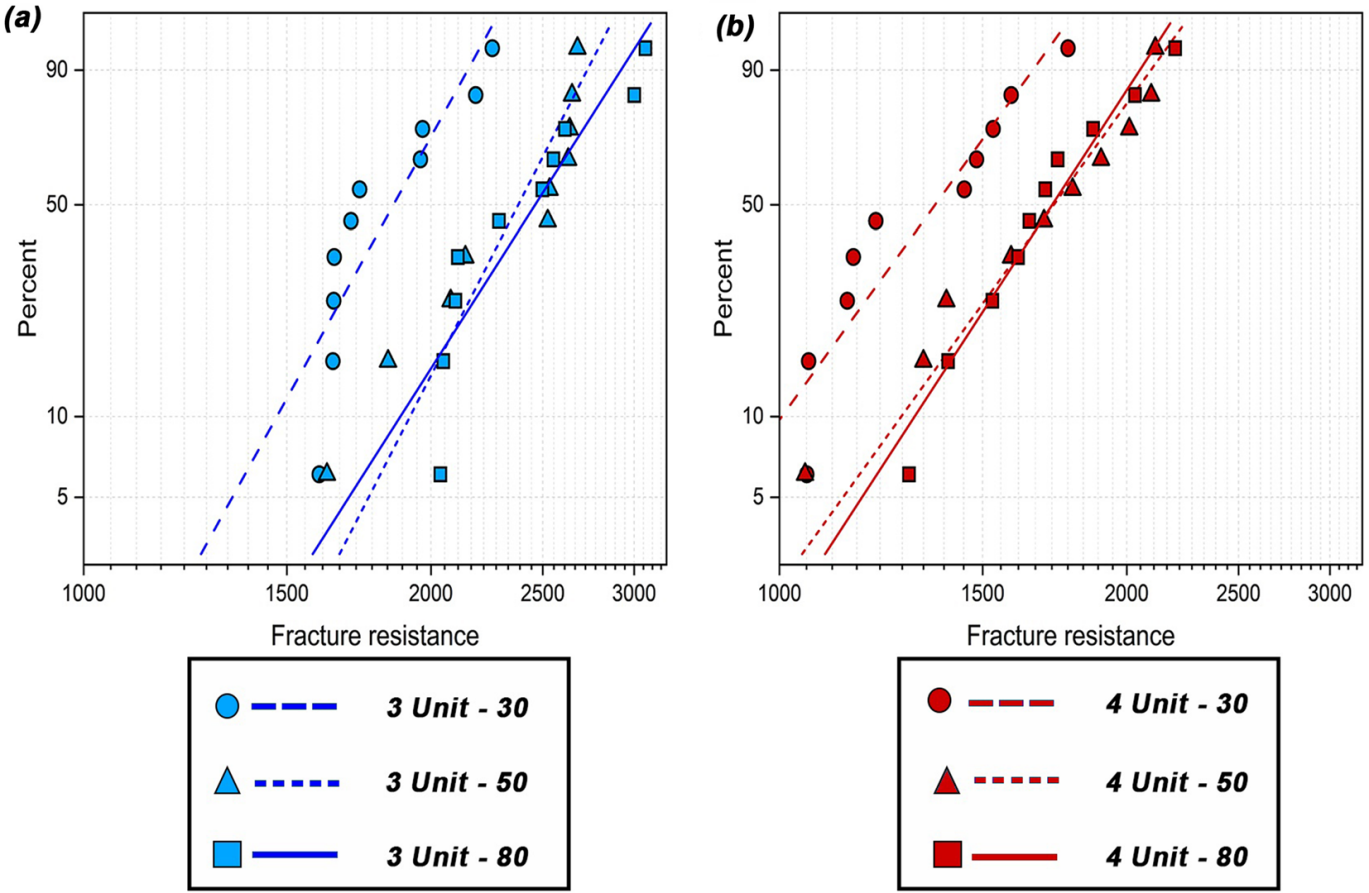
Measuring total cement area with image processing software program. **A**, importing ×25 magnification images to program. **B**, thresholding cement space area. **C**, transforming threshold color into black. **D**, deleting unnecessary data and calculating area limited to threshold color

The zirconia frameworks were assessed for marginal and internal fit accuracy with the replica method [29–32]. For each zirconia framework, the retainers were filled with a light body silicone impression material (Kromopan superlight body; LASCOD S.P.A.) and seated on the corresponding die under a static load of 20 N until the silicone was set [33]. The framework was removed with the silicone layer inside representing the cement area, and filled with a light body silicone material with a contrasting color (Elit HD + light body; Zhermack S.P.A.). The double layered silicone replicas were removed from the retainers and sectioned with a blade into 4 segments. The segmented replicas were assessed under a stereomicroscope (B061; Olympus Japan) to obtain images of die replicas at ×25 and ×40 magnification. The marginal gap, and the internal gap at mid-axial, axio-occlusal, and mid-occlusal areas were measured in µm according to Holmes et al. [29] with an image processing software program (Fiji ImageJ, version 2.14.0; NIH USA) using the ×40 magnification images (Fig. 1). The total cement area was assessed with the software program using the ×25 magnification images of the replicas. After setting the scale, color thresholding was confined to the color representing the cement space. The chosen color threshold was then transformed into black using the binary feature. Only the areas pertaining to the cement space were preserved and any black areas within the same color threshold but unrelated to cement space were deselected. The measurements were set to area and limited to threshold to calculate area of cement space in mm^2^ [34]. Figure 2 displays the steps followed for measuring total cement area with Image J software program.

### Assessment of fracture strength

The zirconia frameworks were cleaned in an ultrasonic bath and dried. All frameworks were cemented to their dies with glass ionomer cement (Ketac-Cem EasyMix; 3MESPE) under a 20 N static load for 10 min at a room temperature of 24 °C and a relative humidity of 50 ± 10% and stored for 48 h in 37 °C distilled water [15, 33]. The cemented frameworks were tested for fracture strength in a universal testing machine (5ST, Tinius Olsen, England). A stainless-steel ball with 5 mm diameter was applied to the central fossa of the pontic of 3-unit frameworks, and two connected balls with 5 mm diameter each were centralized over the two pontics of 4-unit frameworks. Rubber dam sheets of thickness 0.5 mm were applied between the occlusal surface of the pontics and the stainless-steel balls for stress distribution. The load was applied at a crosshead speed of 1 mm/min and the fracture force was recorded in Newton (N) with inbuilt software. The Weibull distribution including Weibull modulus (m) and the characteristic fracture load (σ0) were estimated at 95% CI using Origin software (OriginPro, Version 2024, OriginLab Corp.). The fractured specimens were photographed and assessed for fracture mode.

### Statistical analysis

The results data were statistically analyzed with a statistical package (IBM SPSS Statistics, v24.0; IBM Corp). The Shapiro–Wilk test and Kolmogorov–Smirnov test were used to estimate the normality of the data distribution. Two-way ANOVA was used to detect the significant effect of cement spacer and span length on fit accuracy and fracture strength on the zirconia frameworks. Post hoc Tukey test was used for pairwise comparison between study groups. The significance level was adjusted to α = 0.05 for all statistical tests used.

## Results

Table 1 summarizes the descriptive statistics for marginal, internal, and total cement gaps. The cement spacer had a significant effect on gap values for 3-unit and 4-unit frameworks. For 3-unit frameworks, group 3u-50 had significantly less marginal, internal, and total cement gap (42 ± 13, 100 ± 16, and 86 ± 14 μm) followed by group 3u-80 (58 ± 15, 121 ± 18, and 107 ± 13 μm) and group 3u-30 (94 ± 47, 133 ± 27, and 122 ± 30 μm). For 4-unit frameworks, group 4u-50 and group 4u-80 had no significant difference for marginal gap (53 ± 17 and 63 ± 21 μm, *P* = .27*)*, and both groups had significantly less marginal gap compared to group 4u-30 (109 ± 17 μm). However, group 4u-50 had significantly less internal gap and total cement gap (115 ± 15 and 97 ± 12 μm) followed by group 4u-80 (132 ± 20 and 115 ± 17 μm) and group 4u-30 (142 ± 66 and 126 ± 72 μm). The 3-unit frameworks had significantly better fit accuracy compared to 4-unit frameworks at 50 μm and 80 μm while no significant difference was found at 30 μm spacer (*P* > .05).

**Table 1:** Mean ± SD for marginal, internal, and total cement gap values for study groups in µm

Marginal gap				Internal gap				Total cement gap			
**3u-30**	94 ± 47^a^	**4u-30**	109 ± 17^a^	**3u-30**	133 ± 27^a^	**4u-30**	142 ± 66^a^	**3u-30**	122 ± 30^a^	**4u-30**	126 ± 72^a^
**3u-50**	42 ± 13^b^	**4u-50**	53 ± 17^B^	**3u-50**	100 ± 16^b^	**4u-50**	115 ± 15^B^	**3u-50**	86 ± 14^b^	**4u-50**	97 ± 12^B^
**3u-80**	58 ± 15^c^	**4u-80**	63 ± 21^B^	**3u-80**	121 ± 18^c^	**4u-80**	132 ± 20^C^	**3u-80**	107 ± 13^c^	**4u-80**	115 ± 17^C^

SD, standard deviation. Different superscript letters indicate significant difference between study groups in the same column for each gap value. Capital superscript letters indicate significant difference between study groups in the same row

Table 2 displays the descriptive statistics for total cement area in mm^2^. The cement spacer had a significant effect on total cement area for both 3-unit and 4-unit frameworks. For both 3-unit and 4-unit frameworks, the 50 μm spacer produced significantly less total cement area (1.09 ± 0.25 and 1.24 ± 0.34 mm^2^) followed by 80 μm (1.39 ± 0.27 and 1.51 ± 0.33 mm^2^) and 30 μm spacers (1.67 ± 0.42 and 1.80 ± 0.49 mm^2^). The 3-unit frameworks had significantly less total cement area than 4-unit frameworks at all tested cement spacers.

**Table 2:** Mean ± SD for total cement area in mm^2^ for study groups

**3u-30**	1.67 ± 0.42^a^	**4u-30**	1.80 ± 0.49^A^
**3u-50**	1.09 ± 0.25^b^	**4u-50**	1.24 ± 0.34^B^
**3u-80**	1.39 ± 0.27^c^	**4u-80**	1.51 ± 0.33^C^

SD, standard deviation. Different superscript letters indicate significant difference between study groups in the same column. Capital superscript letters indicate significant difference between study groups in the same row

Table 3 summarizes the descriptive statistics for fracture strength. The cement spacer had a significant effect on fracture strength for both 3-unit and 4-unit frameworks. For both 3-unit and 4-unit frameworks, no significant difference was detected between 50 μm (2334 ± 383.9 and 1698.7 ± 355.7 N) and 80 μm spacers (2432.5 ± 382.4 and 1703.1 ± 276.6 N), and both spacer settings had significantly better fracture strength than 30 μm spacer (1835.5 ± 241.7 and 1346 ± 251.3 N). The 3-unit frameworks had significantly higher fracture strength than 4-unit frameworks at all cement spacers.

**Table 3:** Mean ± SD for fracture resistance in N for study groups

**3u-30**	1835.5 ± 241.7^a^	**4u-30**	1346 ± 251.3^A^
**3u-50**	2334 ± 383.9^b^	**4u-50**	1698.7 ± 355.7^B^
**3u-80**	2432.5 ± 382.4^b^	**4u-80**	1703.1 ± 276.6^B^

SD, standard deviation. Different superscript letters indicate significant difference between study groups in the same column. Capital superscript letters indicate significant difference between study groups in the same row

**Fig. 3:**
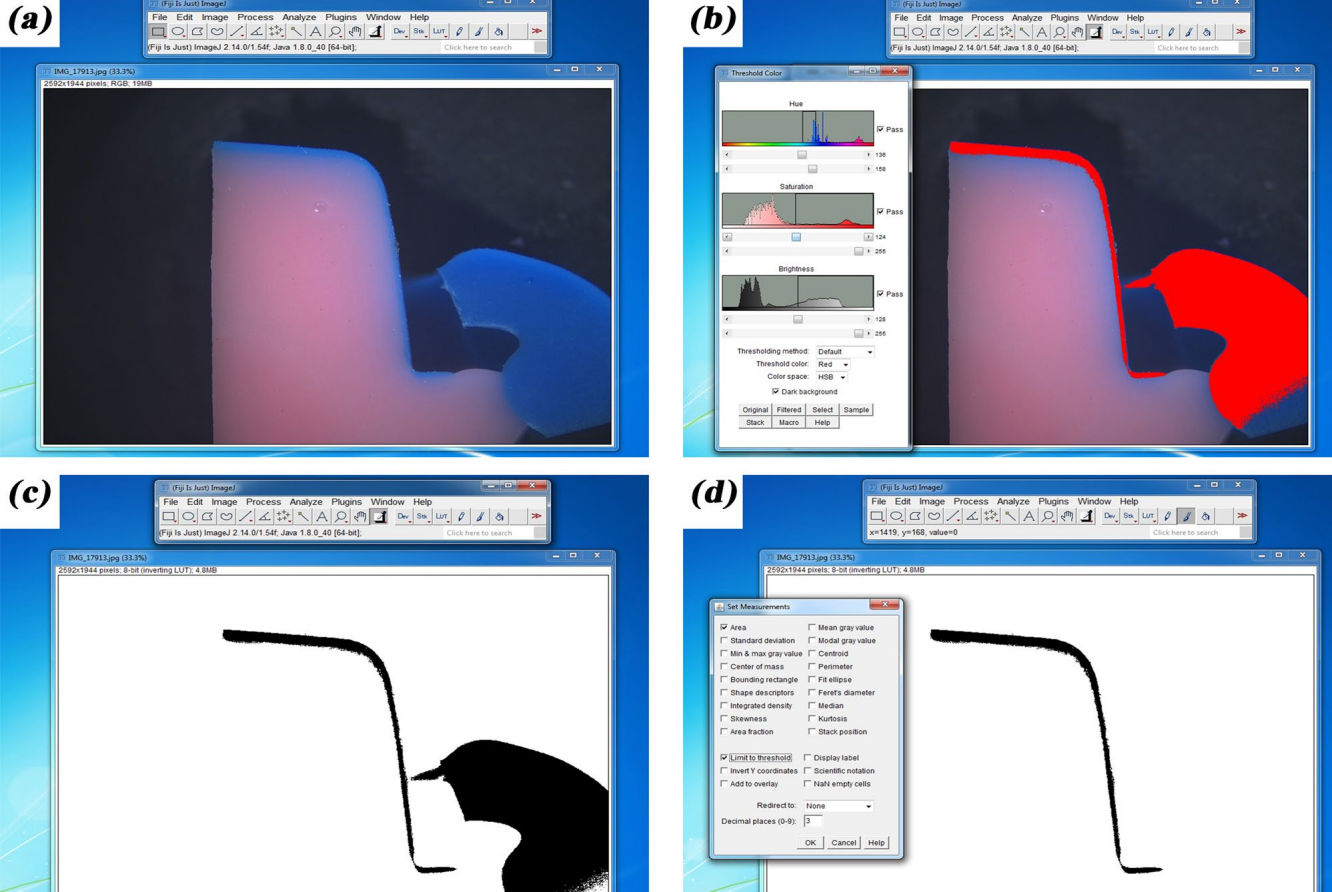
Weibull parameters distribution for study groups

Table 4; Fig. 3 display the Weibull distribution for study groups. No significant difference in the Weibull parameters was found among 3-unit frameworks or among 4-unit frameworks with different spacers (*P* = .13). However, a significant difference was found in both parameters between 3-unit and 4-unit frameworks at the same spacer setting (*P* = .02). The 3-unit frameworks had higher values for Weibull parameters estimate than 4-unit at all spacer settings.

**Table 4:** Weibull distribution for study groups

Study group	Weibull shape (m)				Weibull scale (σ0)			
	Estimate	SD error	Lower	Upper	Estimate	SD error	Lower	Upper
3u-30	8.1004	1.9104	5.1022	12.8602	1941.3454	80.6009	1789.6273	2105.9257
3u-50	8.7198	2.4182	5.0634	15.0164	2482.6101	94.0388	2304.9730	2673.9372
3u-80	7.0065	1.6713	4.3900	11.1825	2593.9967	124.3415	2361.3895	2849.5167
4u-30	6.1571	1.4992	3.8204	9.9230	1447.8626	78.7955	1301.3775	1610.8362
4u-50	6.1903	1.6206	3.7058	10.3406	1834.3256	98.4371	1651.1922	2037.7702
4u-80	6.8606	1.6253	4.3123	10.9148	1817.8877	88.8740	1651.7830	2000.6961

SD, standard

**Fig. 4:**
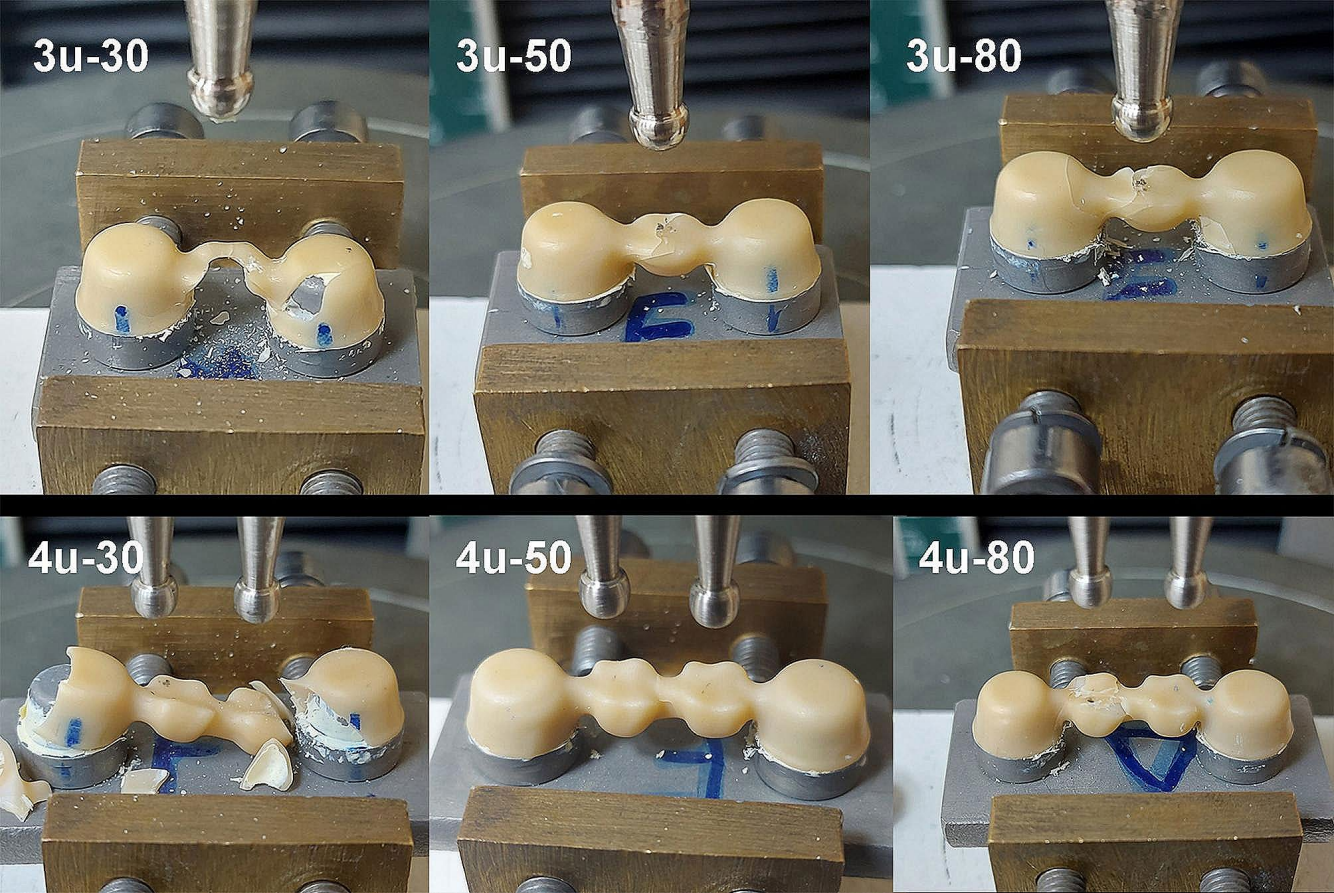
Representative specimens for failure mode of study groups

The mode of failure of 3-unit specimens occurred as cracks at the point of load application. For 3u-30 and 3u-80, the cracks propagated more to the abutments with more incidence of catastrophic failure in group 3u-30. For 4-unit specimens, the cracks originated at the cervical surface of connector and propagated to the abutments and/or the pontic with catastrophic failure in the majority of the specimens in group 4u-30 and 4u-80 and most of the specimens in group 4u-50 (Fig. 4).

## Discussion

This study was conducted to investigate the effect of cement spacer on fit accuracy and fracture strength for 3-unit and 4-unit zirconia frameworks. The null hypothesis was rejected as cement spacer had a significant effect on marginal and internal adaptation and fracture strength of zirconia frameworks. In this study, fit accuracy and fracture resistance were assessed, as both are critical for long term success of fixed restorations, and both can be affected by cement thickness [3–7]. The cement spacer settings assessed in this research were 80 μm, as recommended by the CAD software program manufacturer, 30 μm and 50 μm as recommended settings in the literature [22–27]. Marginal and internal adaptation were assessed in this study with 2 different methods, one is the validated replica method [29–32]. One of the disadvantages of the replica method is the limited measuring points for marginal and internal gaps on the cement layer represented by the silicone replica [35]. The other method for assessment in this research was a modification of the replica technique by using an image analysis software with the same replica images [34]. The advantage of this method is that unlike the conventional replica method the whole total cement area was measured. The results for total cement gap in µm (average of marginal, mid-axial, axio-occlusal, and occlusal gaps) measured with conventional replica method was consistent with total cement area in mm^2^ measured with the modified method in the current study. Consequently, the modified replica method used in this research can be considered as a reliable method for similar future research. Both 3-unit and 4-unit frameworks had significantly better adaptation with a 50 μm cement spacer, followed by 80 μm and 30 μm cement spacers. In addition, the specimens with 50 μm spacer had more even silicone layer than the other spacers. This finding might be attributed to sintering shrinkage and deformation of zirconia frameworks that could not be compensated with a 30 μm cement spacer and lead to improper seating and poor fit accuracy [3, 6]. On the other side, an 80 μm cement spacer might have created excess space for cement (silicone), increased hydrodynamic pressures, and improper seating and adaptation of the frameworks [36]. Previous studies reported a marginal gap mean value for 3-unit zirconia FPDs ranging between 20 ± 5 μm and 106 ± 45 μm, a mean internal gap value between 30 ± 13 μm and 134 ± 47 μm [9, 12–14]. For 4-unit zirconia FPDs, Similar studies reported a marginal gap mean value for 4-unit zirconia FPDs ranging between 63 ± 36 μm and 141 ± 193 μm, a mean internal gap value between 58 ± 35 μm and 165 ± 137 μm [6, 11, 18]. The results for marginal and internal fit in the current research agree with Grajower and Lewinstein [37], who recommended the 50 μm spacer for better fit accuracy of fixed restorations. In addition, the results of the current research are consistent withKale et al. [22], and Schriwer et al. [23], who reported that the 30 μm spacer jeopardized the fit accuracy of CAD-CAM zirconia.On the other hand, the results of this study disagree with Suzuki et al. [27] who reported better adaptation with 30 μm spacer compared to 45 μm. However, the spacer settings assessed in this study were different.

In the current research, fracture resistance was assessed by measuring failure load and running the Weibull statistics to obtain more reliable results [7, 16]. Before failure load testing, the specimens were cemented with glass ionomer cement as performed in previous similar studies to simulate clinical routine [7, 7–17, 19, 20]. For fracture strength, both 50 μm and 80 μm had similar results, and both had significantly better fracture strength compared to 30 μm spacer for 3-unit and 4-unit specimens. This might be attributed to the ununiform cement layer with the 30 μm spacer that increased sintering stresses concentration within the specimens [4, 5]. The results for failure load in this study were within the range of the values reported in the literature [7–17, 19, 20]. The findings of this study are consistent with Rezende et al. [5], who reported that thicker cement space associated with misfit increased stress concentrations for zirconia crowns. On the other hand, the results of this study disagree with Schriwer et al. [23], who reported no significant effect for cement space on fracture strength of zirconia crowns. Unfortunately, the authors are unaware of available studies on the effect of cement spacer on zirconia FPDs to compare with the obtained results in the current research.

In Weibull statistic, shape parameter (m) indicates the predictability of fracture as a result of material flaws or defects, while scale parameter (σ0) indicates the characteristic fracture strength for 63.21% of the specimens [38]. No significant difference was found in the Weibull parameters between different spacers for 3-unit and 4-unit specimens. The Weibull distributions displayed a considerable overlap between 50 μm and 80 μm specimens, which was consistent with the results for fracture strength. The 3-unit specimens had significantly higher Weibull parameters than 4-unit specimens regardless of spacer setting, which agreed with the fracture strength results.

The results obtained in this research for Weibull parameters were consistent with the values reported in the literature [7, 16]. Similarly, the mode of failure in this study is consistent with the literature where the fracture initiated at the connectors or at the point of load application [4, 7, 15–20]. The mode of failure of specimens reflects the results of internal adaptation where the 30 μm and 80 μm spacers produced thick and uneven cement layer with more stress distribution and crack propagation within the axial walls.

For both adaptation and fracture resistance, 3-unit frameworks produced significantly better results compared to 4-unit frameworks regardless of spacer settings. As a result of increased volume of zirconia for 4-unit specimens, the sintering shrinkage and deformation might have increased with decreased adaptation and fracture resistance compared to 3-unit specimens [11].

The limitations of this study include the steel master dies used, which did not simulate dentin resiliency. However, this set up was selected for the purpose of standardization which would be difficult with natural teeth as abutments. In addition, the small sample size, as the fracture of dental ceramics is probabilistic, a larger sample size can be useful to reach more reliable results for future investigations. In addition, future research on monolithic zirconia FPDs with different cement spacer settings is recommended.

## Conclusions

1. Both cement spacer and span length had a significant effect on fit accuracy and fracture strength of zirconia frameworks.
2. A cement spacer of 50 μm had significantly better marginal and internal fit followed by 80 μm and 30 μm spacers for both 3-unit and 4-unit zirconia frameworks.
3. The 50 μm and 80 μm spacers produced similar fracture strength, and both produced significantly better strength than 30 μm spacer for both 3-unit and 4-unit zirconia frameworks.
4. The 3-unit zirconia frameworks had significantly better adaptation and fracture strength compared to 4-unit frameworks regardless of the spacer setting used.
5. The 50 μm is recommended for fabricating both 3-unit and 4-unit zirconia frameworks with satisfactory fit accuracy and fracture strength.

## Data Availability

The datasets used and/or analysed during the current study are available from the corresponding author on reasonable request.

## Abbreviations

CAD-CAM: Computer-aided designing-computer-aided milling
FPD: Fixed partial denture
3u: 3-unit
4u: 4-unit
